# Clear cell odontogenic carcinoma: a diagnostic and therapeutic dilemma

**DOI:** 10.1186/1477-7819-4-91

**Published:** 2006-12-12

**Authors:** Singh Avninder, Dinesh Rakheja, Amar Bhatnagar

**Affiliations:** 1 Institute of Pathology-ICMR, Safdarjung Hospital Campus, New Delhi, India; 2 Department of Pathology, University of Texas Southwestern medical center, Dallas, Texas, USA; 3 Department of Cancer Surgery, Safdarjung Hospital, New Delhi, India

## Abstract

**Background:**

Clear cell odontogenic carcinoma is a rare odontogenic tumor occurring in the anterior region of the mandible in 5^th^–7^th ^decades and shows a female preponderance. It is potentially aggressive, capable of frequent recurrences and loco-regional and distant metastases.

**Case presentation:**

A 45- year- old woman presented with a radiolucent left mandibular swelling associated with loss of teeth. Left cervical lymph nodes were enlarged on palpation. The patient underwent resection of the tumor but consequent to resected margins being positive for tumor cells underwent left hemimandibulectomy with ipsilateral functional neck dissection and was free of recurrence at 8 months follow-up.

**Conclusion:**

Clear cell odontogenic carcinoma should be considered in the differential diagnosis of jaw tumors with conspicuous clear cell component. Curettage or conservative resection inevitably results in recurrences and/or metastasis and more radical resection is warranted in these tumors, especially when they are large and show soft tissue invasion.

## Background

Clear cell odontogenic carcinoma (CCOC) is a rare odontogenic tumor arising from the anterior region of mandible and has a predilection for females with 43 cases reported in literature. We report a case of CCOC in the mandible of a 45- year- old female with lymph node metastasis

## Case presentation

**Figure 1:**
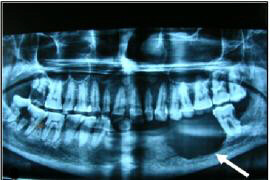
OPG showing a radiolucent lesion with irregular bony margins, partly destroying the left mandibular body (arrow).

A 45-year-old woman presented with a painful swelling on the left side of her jaw of 5 months duration. The swelling had recently started to grow rapidly. There were no associated sensory symptoms. In addition, a few teeth adjacent to the swelling had become loose, carious and had fallen off. Local physical examination showed a 4.0 × 3.2 × 3.0 cm, tender, firm, irregular lump that appeared to arise from the left mandible. Intra orally, the tumor could be seen as a pink, bulging and fleshy mass. Two lymph nodes were palpable, one in left submaxillary region measuring approximately 1 × 1 cm and the other in the left cervical region and was barely palpable. Remainder of the physical examination was not contributory and no other swelling was noticed. Orthopantomogram (OPG) of the jaw showed a radiolucent lesion with irregular margins, centered in and destroying a portion of the left mandibular body (Figure 1). The teeth adjacent to the mass were carious and some were missing. A computed tomography (CT) scan of the oral cavity and neck was advised but was not done due to financial reasons. A clinical diagnosis of ameloblastoma with suspicion of lymph node involvement was given.

### Pathological findings

**Figure 2:**
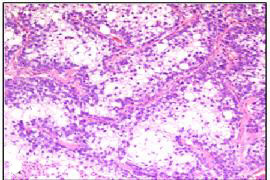
Section showing central clear cells separated by fibrovascular septae (H&E, ×100).

**Figure 3:**
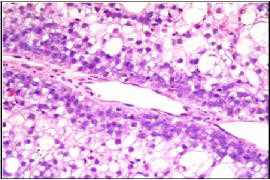
Section showing palisaded cells with reverse nuclear polarity (H&E, ×400).

**Figure 4:**
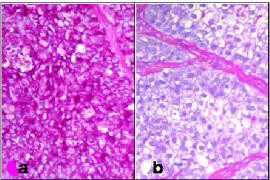
Section showing (a) PAS positive and (b) diastase labile tumor cells (H&E, ×100).

An incisional biopsy of the lesion was submitted for pathologic evaluation. Microscopic examination of the tissue showed a neoplasm composed of epithelial cells arranged in irregular nests separated by fibrovascular septa. The cells adjacent to the fibrovascular septa were cuboidal to columnar with high nuclear-cytoplasmic ratio and eosinophilic cytoplasm, while those in the center of the nests were larger and polygonal, with abundant clear cytoplasm (Figure 2). Each cell had a single nucleus with fine chromatin and prominent eosinophilic nucleolus. The cells at the periphery of the nests occasionally demonstrated nuclear palisading away from the basement membrane, i.e., reverse nuclear polarity (Figure 3). These peripheral cells also showed occasional mitoses. The central areas of the nests and fibrous septae showed infiltration by neutrophils. The cells were immunoreactive for pan-cytokeratin, epithelial membrane antigen, and S-100 protein; they did not stain for vimentin, smooth muscle actin, and HMB-45. The abundant, clear cytoplasm of the cells was strongly positive for periodic acid-Schiff (PAS) (Figure 4a). This PAS positivity was diastase sensitive indicating intracytoplasmic glycogen (Figure 4b). A diagnosis of clear cell odontogenic carcinoma was entertained. Fine needle aspiration from the submaxillary node was done but was inconclusive.

The patient was referred to a specialty cancer hospital where she underwent left hemimandibulectomy with ipsilateral functional neck dissection (level1 to level 5). The reconstruction was done using ipsilateral 12th rib and the soft tissue coverage was given by pectoralis major flap. Postoperative histopathological examination showed tumor morphology similar to incisional biopsy and 1/11 lymph nodes (submaxillary) was positive for the tumor metastasis without any extranodal spread. The tumor was extending into the soft tissue and the left bony margin was positive for the tumor cells. Due to the positive margin, the patient was referred for adjuvant radiotherapy for which she refused to give consent. At the time of discharge the patient was accepting soft diet and the suture line on the neck and chest were healthy. Subsequent to the discharge, the patient was lost to follow-up only to reappear after 8 months and at that time had no signs of local or regional spread. Since then patient is again lost to follow-up.

## Discussion

In 1985, Hansen *et al*., reported a locally aggressive odontogenic neoplasm, and named it clear cell odontogenic tumor [1]. This neoplasm was initially thought to be devoid of malignant potential and classified as benign [2]. Subsequent reports of their aggressive behavior, predilection for local recurrence, evidence of pulmonary and lymph node metastases and tumor-related deaths necessitated a change in their classification and nomenclature and is now called CCOC [3-7].

The classic clinical presentation of CCOC is a painful anterior mandibular swelling in an elderly woman. There may be loosening of adjacent teeth, and a roentgenogram shows an irregular radiolucent mass, most often in a pre-molar location. Histopathologically, CCOCs may show one or more of three architectural patterns: biphasic, monophasic, and ameloblastomatous. The most common biphasic pattern of tumor growth comprises of nests of cells with clear cytoplasm admixed with cells containing eosinophilic cytoplasm. The monophasic pattern comprises only of clear cells, while the ameloblastomatous pattern resembles the growth pattern of ameloblastoma with nests of cells showing central cystic change and squamous differentiation, and peripheral nuclear palisading with reverse polarity [7]. The differential diagnosis of jaw tumors with prominent cytoplasmic clearing includes intraosseous salivary gland tumors (epithelial-myoepithelial carcinoma, in which the clear myoepithelial cells are immunoreactive for S-100 protein, vimentin, smooth muscle actin, and calponin; mucoepidermoid carcinoma, distinguished by its triphasic architecture comprised of mucin-positive mucous cells, squamoid cells, and intermediate cells) and metastatic tumors (classic clear cell renal cell carcinoma, which can be identified by its characteristically rich vascular pattern and its immunoreactivity for cytokeratins and vimentin and lack of reactivity for S-100 protein; and amelanotic melanoma, which reacts for HMB-45, S-100 protein and other melanoma markers). Other odontogenic tumors may also show clearing of their constituent cells. Such tumors include calcifying epithelial odontogenic tumor and clear cell ameloblastoma. While the former is identified by the presence of psammomatous calcifications and amyloid deposits, the latter may be difficult to distinguish from CCOC. In fact, some authors think that clear cell ameloblastomas and CCOCs may represent a clinico-pathological continuum of a single neoplastic entity [8].

A recent review of literature of the 43 cases reported so far [9] showed that the male: female ratio was 3:1, mean age at presentation was 58 years (range 17–89 years), the average period of follow-up was 5.5 years (range 0.5–21 years), mandible was the most favored location (84%). The overall recurrence rate for these tumors was 55% and local recurrence rates were higher (80%) for curettage alone than for resection alone (43%). Lymph node metastasis on initial presentation was rare (10%) but rapidly increased in those with recurrent disease (33%). Factors such as size of the lesion, soft tissue involvement, lymph node metastasis and most importantly, the presence or absence of positive surgical margins should be considered when developing the treatment strategy.

## Conclusion

CCOC although rare, should be considered in differential diagnosis of jaw tumors with prominent clear cell component. The limited number of reported cases precludes a definitive assessment of prognostic factors or efficacy of therapy in CCOC. Currently, treatment is aimed at achieving wide surgical resection with tumor-free margins, and loco-regional control by lymph node resection and local radiation in cases with extensive soft tissue invasion, perineural spread, lymph node metastasis with extranodal involvement or in those where tumor-free margins are not possible. A long-term follow-up is essential as these tumors may recur locally or present with late distant metastases.

## Competing interests

The author(s) declare that they have no competing interests.

## Authors' contributions

**SA **participated in histopathological diagnosis, reviewed the literature and partly wrote the manuscript. **DR **reviewed the slides, did special stains, took pictures and contributed suggestions in drafting the manuscript. **AB **clinically managed the patient and helped in preparing the draft manuscript. All the authors have read and approved the final version of the manuscript.
